# The Effect of Dual-Layer Carbon/Iron-Doped Buffers in an AlGaN/GaN High-Electron-Mobility Transistor

**DOI:** 10.3390/mi16091034

**Published:** 2025-09-10

**Authors:** Po-Hsuan Chang, Chong-Rong Huang, Chia-Hao Liu, Kuan-Wei Lee, Hsien-Chin Chiu

**Affiliations:** 1 Department of Electronic Engineering, I-Shou University, Kaohsiung 840, Taiwan; vicsjob0612@gmail.com; 2 Department of Electronic Engineering, Chang-Gung University, Taoyuan 333, Taiwan; gain525252@gmail.com (C.-R.H.); r3287133@gmail.com (C.-H.L.)

**Keywords:** GaN, high-electron-mobility transistor (HEMT), carbon (C), iron (Fe)

## Abstract

This study compared the effectiveness of gallium nitride (GaN) with a single carbon-doped (C-doped) buffer layer and a composite carbon/iron-doped (C/Fe-doped) buffer layer within an AlGaN/GaN high-electron-mobility transistor (HEMT). In traditional power devices, Fe-doping has a large memory effect, causing Fe ions to diffuse outwards, which is undesirable in high-power-device applications. In the present study, a C-doped GaN layer was added above the Fe-doped GaN layer to form a composite buffer against Fe ion diffusion. Direct current (DC) characteristics, pulse measurement, low-frequency noise, and variable temperature analysis were performed on both devices. The single C-doped buffer layer in the AlGaN/GaN HEMT had fewer defects in capturing and releasing carriers, and better dynamic characteristics, whereas the composite C/Fe-doped buffers, by suppressing Fe migration toward the channel, showed higher vertical breakdown voltage and lower sheet resistance, and still demonstrated potential for further performance tuning to achieve enhanced semi-insulating behavior. With optimized doping concentrations and layer thicknesses, the dual-layer configuration offers a promising path toward improved trade-offs between leakage suppression, trap control, and dynamic performance for next-generation GaN-based power devices.

## 1. Introduction

Gallium nitride (GaN) high-electron-mobility transistors (HEMTs) are used extensively in high-power and high-frequency applications because of their wide energy band gap, thermal conductivity, and electron saturation speed [1], but problems with reliability persist. These problems stem from the structural design of the unintentionally doped (UID) GaN buffer layer in AlGaN/GaN devices [2]. Specifically, lattice mismatch between the buffer and substrate, nitrogen vacancy, or oxygen impurity within conventional GaN buffer layers creates an unwanted leakage path, resulting in defects and dislocations that are damaging to the device [3]. Some studies have used extrinsic dopants to improve buffer insulation and suppress defects, such as magnesium (Mg), C, and Fe [4,5,6].

Fe is the most commonly used buffer dopant and has a strong memory effect, causing Fe ions to diffuse into the channel layer, limiting device output power and reliability [7,8]. When the Fe-doped concentration is too low, the buffer loses its semi-insulating characteristics, whereas a high concentration causes current collapse within the device [9,10]. Fe creates deep-level traps that compensate n-type carriers and enhance buffer resistivity when appropriately dosed. With Fe properly dosed and confined within the buffer, Fe-induced compensation suppresses parasitic conduction, thereby reducing leakage paths and homogenizing the electric field distribution under high drain bias. Although some studies have shown that C-doped buffers lack memory effect, making them highly resistive and semi-insulating [11,12], others have found that current collapse is more likely when a C-doped buffer is used in GaN HEMTs than when an Fe-doped buffer is used [13,14]. Moreover, although a thicker C-doped GaN buffer layer can improve the off-state breakdown voltage of high-frequency power switching [15], Uren et al. noted a floating C-doped buffer, which a simulation indicated could pinch the channel [2]. An epitaxial back barrier AlGaN has been reported to effectively inhibit dopant diffusion in some studies, but the optimal thermal dissipation, thickness, location, and Al mole fraction remain unclear [3,16].

Limited research has used both Fe and C-doped buffers [16,17] to form a composite structure without a back barrier. In the present study, a composite buffer structure combining a C-doped GaN layer atop an Fe-doped GaN layer is proposed to suppress Fe ion diffusion. The dual-layer structure is experimentally compared with a conventional single C-doped buffer in AlGaN/GaN HEMTs.

## 2. Experimental

**Figure 1:**
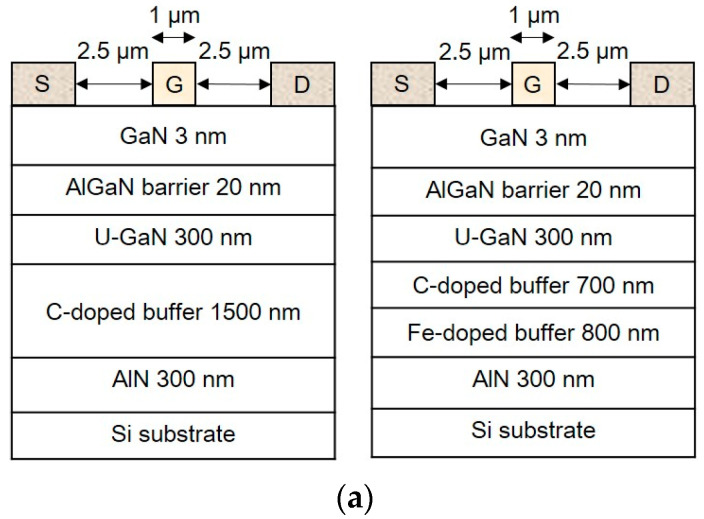

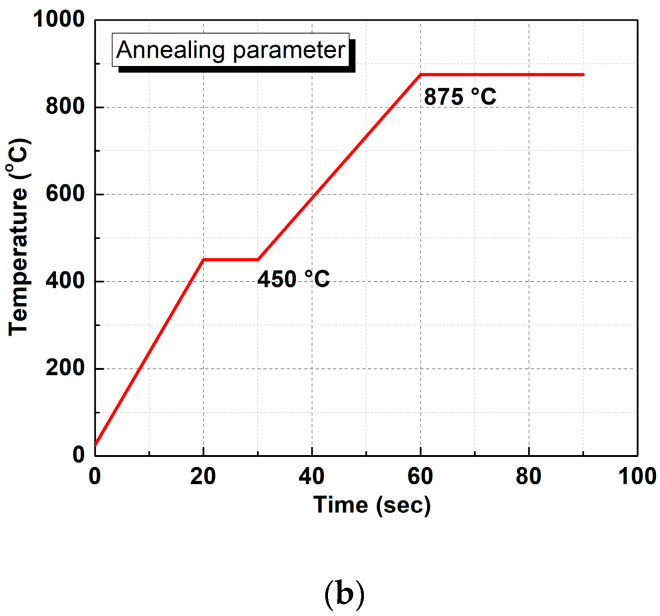
(**a**) AlGaN/GaN HEMTs with single C-doped and composite C/Fe-doped buffer structures and (**b**) RTA parameter diagram.

Two proposed structures for the AlGaN/GaN HEMT were grown using metal–organic chemical vapor deposition (MOCVD) on a silicon (Si) substrate. For MOCVD process, TMGa, TMAl, and NH_3_ were used as the sources of Ga, Al, and N, respectively. Firstly, an initial AlN nucleation layer was grown at 550 °C. An Fe-doped GaN layer was grown at 980 °C, followed by the growth of the periodically carbon-doped GaN buffer layer. Then, intrinsically doped GaN was grown at 1000 °C. The GaN channel, AlN, AlGaN, and GaN cap were all grown under the same pressure conditions and temperature of 100 mbar and 1040 °C, respectively. From bottom to top, the devices consist of a 300 nm AlN epitaxial nucleation layer, a 1500 nm GaN buffer layer, a 300 nm GaN channel layer, a 20 nm AlGaN barrier layer, and a 3 nm GaN cap layer. Figure 1a shows the proposed structures, including one with a single C-doped GaN buffer layer and another with a composite C/Fe-doped GaN buffer layer. Hall measurements indicated that the room-temperature electron mobilities of the devices with a single C-doped buffer and C/Fe-doped buffer are 1.22 × 10^3^ cm^2^/V·s and 1.13 × 10^3^ cm^2^/V·s, respectively. The corresponding carrier densities are 1.84 × 10^13^ cm^−2^ and 2.05 × 10^13^ cm^−2^, respectively. Secondary ion mass spectrometer (SIMS) analysis indicated that the C and Fe concentrations in buffer layers are 1 × 10^19^ cm^−3^ and 2 × 10^18^ cm^−3^, respectively.

Firstly, samples were cleaned to remove surface particles and organic matter by acetone, isopropyl alcohol (IPA), and deionized water for 3 min, respectively. Then they were placed into diluted hydrochloric acid to remove the surface oxides. A reactive ion etching (RIE) system filled with Cl_2_/BCl_3_/Ar plasma was used to define the mesa pattern. Ohmic contacts composed of a 175 nm thick Ti/Al/Ni/Au alloy were deposited by an electron beam (E-beam) evaporator and then patterned using lift-off processes followed by rapid thermal annealing (RTA) in an N_2_ environment (Figure 1b). The gate metal was deposited by lift-off processes with 72.5 nm thick Ni/Au alloy using an E-beam evaporator. To avoid damaging the samples during probe measurement, a metal interconnection composed of a 72.5 nm thick Ti/Au alloy was deposited by an E-beam evaporator on the active regions of the samples. Gate dimension and source-drain spacing for each finger were 1 × 100 μm^2^ and 6 μm, respectively. The direct current (DC) of the devices was measured using Agilent 4142. The dynamic characteristics were measured using the AM213 gate measurement module and AM241 drain measurement module with the AMCAD pulse measurement system. The low-frequency spectral density of drain current noise (S_ID_) was measured using an Agilent N9010A vector signal analyzer with an Agilent 35670A dynamic signal analyzer.

## 3. Results and Discussion

**Figure 2:**
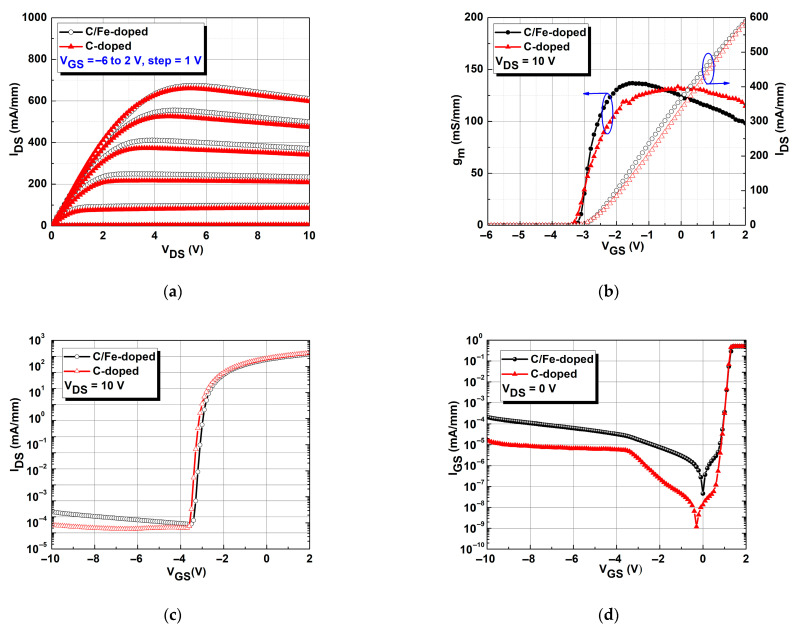
Comparisons of (**a**) I_DS_-V_DS_ curve, (**b**) transfer characteristics at V_DS_ = 10 V, (**c**) subthreshold characteristics, and (**d**) I_GS_-V_GS_ two-terminal diode characteristics for the devices with C/Fe-doped and single C-doped buffers.

Figure 2a displays the drain-to-source current (I_DS_) versus drain-to-source voltage (V_DS_) of the devices, respectively, containing a C/Fe-doped buffer and a single C-doped buffer. Measurement conditions ranged from V_DS_ = 0 to 10 V at intervals of 1 V. On-resistance (R_on_) was calculated using a curve for a gate-to-source voltage (V_GS_) of 2 V. The R_on_ values of the composite C/Fe-doped and single C-doped buffers were 4.97 and 4.69 Ω∙mm, respectively. The reason for this will be explained later in Figure 4. Figure 2b presents the transconductance (g_m_) and I_DS_ with V_GS_ at a fixed V_DS_ = 10 V for both devices for comparison. When V_GS_ = 2 V, the maximum I_DS_ values for C/Fe-doped and C-doped devices were 594 and 583 mA/mm, respectively, with corresponding peak g_m_ values of 139.7 and 134.7 mS/mm, respectively. Figure 2c shows the subthreshold characteristics for both devices. The subthreshold swing (*SS*) of the C/Fe-doped buffer (123 mV/dec) was slightly higher than that of the C-doped buffer (107 mV/dec), clearly indicating that unwanted off-state electron flow was reduced by one order of magnitude for the device with the C-doped buffer compared with that containing the C/Fe-doped buffer. The related I_ON_/I_OFF_ ratios were 6.71 × 10^5^ and 1.13 × 10^6^, respectively. I_ON_ was defined as I_DS_ at V_GS_ = threshold voltage + 1 V, and I_OFF_ was defined as I_DS_ at V_GS_ = threshold voltage − 1 V. Improved *SS* and I_ON_/I_OFF_ ratios are related to the reduced leakage current. Figure 2d shows the two-terminal diode characteristics of I_GS_ against V_GS_ when V_DS_ = 0 V [18] for the V_GS_ range of −10 to 2 V. When V_GS_ = −10 V (i.e., off-state), the gate leakage current of the C/Fe-doped buffer was 2.06 × 10^−4^ mA/mm, which was higher than that of the C-doped buffer (1.79 × 10^−5^ mA/mm). Although both devices had similar DC characteristics, they exhibited noticeable differences in leakage current density [19]. Reduced leakage current density was due to the doping agent creating a more resistive buffer [20].

**Figure 3:**
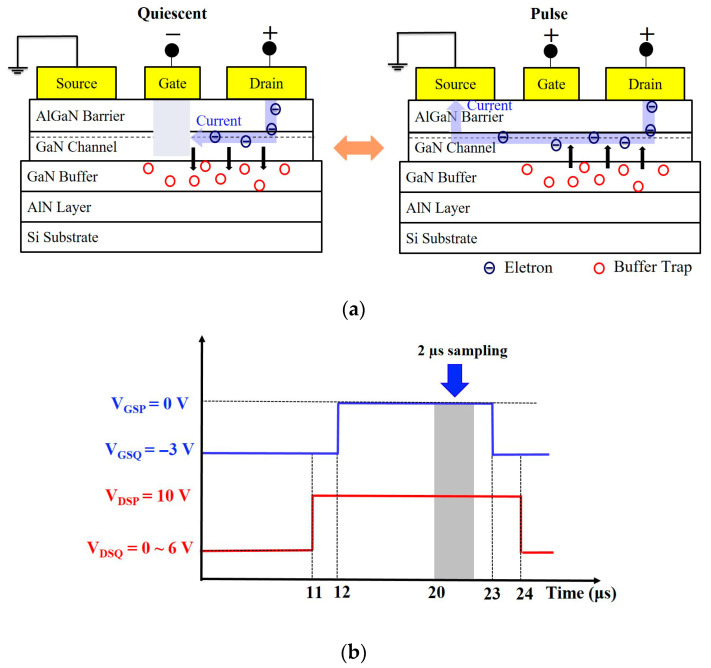
(**a**) Trapping and release of electrons by buffer defects between quiescent and pulse biases. (**b**) Dynamic measurement conditions.

In AlGaN/GaN HEMTs, defects mainly appear on the semiconductor surface, the interface near the two-dimensional electron gas (2DEG) channel, and the epitaxial buffer layer. Our study focused on extrinsic dopants in the buffer layer, using pulses to measure current collapse and analyze drain lag behavior, the latter of which is mainly caused by buffer defects. When a bias voltage is applied, buffer defects can trap electrons, causing them to not be released in time. Consequently, the device fails to reach the expected operating current immediately after being turned on, and the additional time taken to increase the output voltage results in poor switching characteristics [21], as shown in Figure 3a. Figure 3b provides the measurement conditions of the drain lag for both devices, which were set as follows: the time period for measurement was 200 μs, the pulse width was 2 μs, the pulse biases were V_GSP_ = 0 V and V_DSP_ = 10 V, and the static biases were V_GSQ_ = −3 V and V_DSQ_ = 0 to 6 V. This figure only displays the conditions for a single on–off cycle of the device. However, overall pulse measurement involved constantly switching the device on and off. The drain was given a positive bias when the device was switched off to allow for observation of electron response and the influence of drain lag behavior. Therefore, both devices were switched off throughout pulse measurement, with static bias points set as (V_GSQ_ = 0 V, V_DSQ_ = 0 V), (V_GSQ_ = −3 V, V_DSQ_ = 1 V), (V_GSQ_ = −3 V, V_DSQ_ = 2 V), (V_GSQ_ = −3 V, V_DSQ_ = 3 V), (V_GSQ_ = −3 V, V_DSQ_ = 4 V), (V_GSQ_ = −3 V, V_DSQ_ = 5 V), and (V_GSQ_ = −3 V, V_DSQ_ = 6 V).

**Figure 4:**
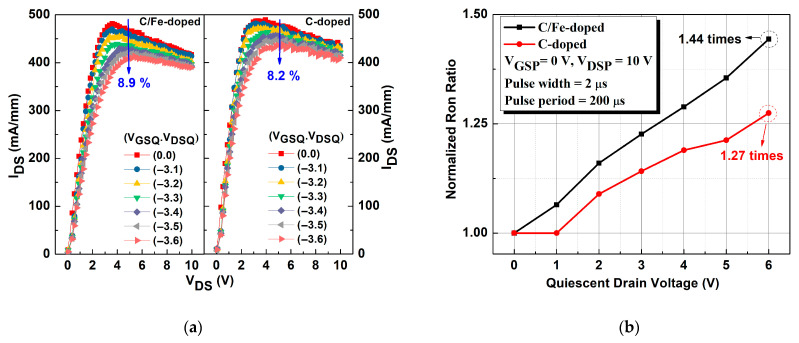
(**a**) Pulse measurement for determining I_DS_-V_DS_ curves for both devices. (**b**) Dynamic R_on_ ratio for both devices.

Figure 4a shows pulse measurement results for both devices, indicating that current collapse decreased by 8.9% in the device with a C/Fe-doped buffer, which was higher than the decrease observed in the device with the C-doped buffer (8.2%), meaning that more electrons were trapped in the C/Fe-doped buffer due to defects. We obtained the I_DS_-V_DSP_ curves of both devices and converted the ratio of the dynamic resistance with the current in the linear region, as shown in Figure 4b. Increased static voltage (V_DSQ_) led to an increased dynamic resistance ratio in the device with a single C-doped buffer, which also exhibited a dynamic R_on_ (1.27 times) lower than that of the C/Fe-doped buffer (1.44 times) at V_DSQ_ = 6 V. This result indicates that defects in the single C-doped buffer had a lower probability of trapping electrons and better dynamic resistance characteristics.

**Figure 5:**
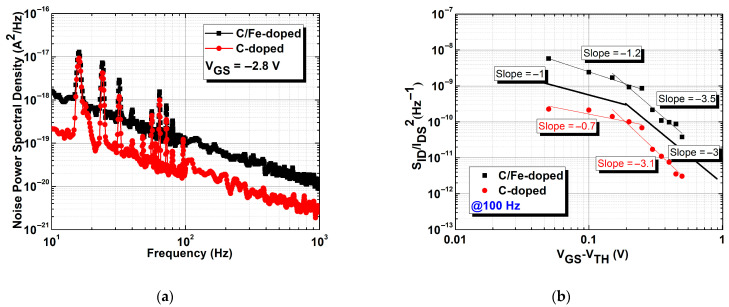
(**a**) Comparison of low-frequency noise spectra at V_GS_ = −2.8 V. (**b**) Gate voltage dependence of the S_ID_/I_DS_^2^ at 100 Hz for both devices.

To understand the trapping and de-trapping caused by buffer defects, low-frequency noise spectra of both devices were measured at −2.8 V of V_GS_ from 10 Hz to 1000 Hz, as shown in Figure 5a. The S_ID_ values for the C/Fe-doped buffer were higher than those for the single C-doped buffer at all frequencies. Both devices are dominated by 1/f noise from ~10 Hz to the mid-frequency range, consistent with a broad distribution of trap time constants. On top of this 1/f background, the C/Fe-doped buffer exhibits Lorentzian-like components—segments where the slope approaches 1/f^2^ beyond a corner frequency, indicating contributions from specific traps. The apparent knees occur at higher frequencies for the C/Fe-doped sample (~100 Hz) than for the C-doped sample (ten of Hz), implying trap time constants of ~1.5 ms and ~4.0 ms, respectively. A lower corner frequency indicates a longer trap response time and is generally associated with deeper traps or fewer trap states, as in the C-doped buffer. Conversely, the higher corner frequency of the composite C/Fe-doped buffer suggests shorter trap time constants, which may result from shallower energy levels introduced by Fe doping. To locate the noise source in both devices, S_ID_/I_DS_^2^ against V_GS_ at 100 Hz was plotted, as shown in Figure 5b: V_GS_ increased from the threshold voltage (−2.8 V), and S_ID_/I_DS_^2^ exhibited a downward trend with the gradient of both slopes close to −1 and −3 [22]. V_GS_^−1^ was associated with noise from the interface near the gate region, and V_GS_^−3^ was associated with noise caused by channel and buffer defects [17,23]. Results indicated that the C/Fe-doped buffer had a higher proportion of noise caused by buffer defects at the V_GS_^−3^ region, leading to the obvious capture and release of carriers, which is consistent with the results of the drain lag assessment shown in Figure 4.

**Figure 6:**
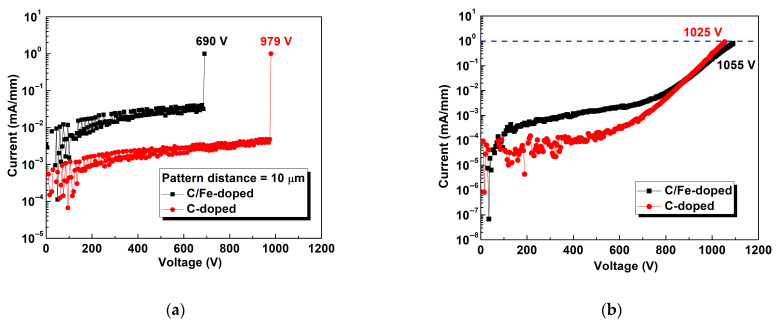

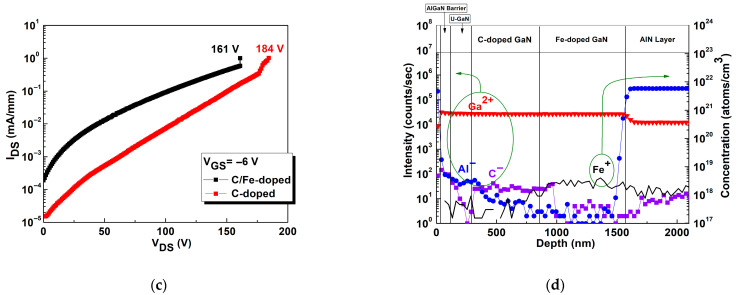
Comparison of (**a**) two-terminal horizontal breakdown voltage measurement, (**b**) two-terminal vertical breakdown voltage measurement, and (**c**) off-state breakdown voltage measurement for both devices. (**d**) SIMS analysis of C/Fe-doped buffer.

Figure 6a shows the two-terminal horizontal breakdown voltages for both devices, for which contact pattern distance was 10 μm. The measured current flowed through the buffer layer, and this allowed us to analyze the level of isolation between both active regions. The horizontal breakdown voltages were 979 and 690 V for the devices with single C-doped buffer and composite C/Fe-doped buffer, respectively. The energy level of the Fe-doped GaN buffer was approximately 0.5–0.6 eV below the bottom of the conduction band. The energy level of the C-doped GaN buffer was approximately 0.9 eV above the top of the valence band [24]. C doping offers better process control but has a great impact on current collapse effect, while Fe doping tends to affect the electrons in the channel more due to the diffusion effect. Thus, the Fe-doped buffer had less of a semi-insulating effect than the C-doped buffer, leading to a larger leakage current or smaller breakdown voltage.

During our experiment, after the cap layer was etched, the metal was plated and the device was secured onto the copper plate using silver glue. We then increased the voltage to determine the maximum voltage that the buffer and substrate could withstand. Figure 6b shows the vertical breakdown voltages, which were 1025 and 1055 V for the devices with the single C-doped buffer and C/Fe-doped buffer, respectively. The behavior of current at voltages below 800 V was due to the resistance in the buffer layer, whereas that of current above 800 V was due to the resistance of the substrate. The crossover may have happened at approximately 800 V because of differences in the resistance of Si substrates produced during epitaxy. Because substrates came from the same batch, differences in breakdown voltage were minimal past 800 V. Figure 6c compares off-state breakdown voltage measurement at V_GS_ = −6 V for both devices. The off-state breakdown voltages were 184 and 161 V for the devices with the single C-doped buffer and composite C/Fe-doped buffer, respectively. It can be seen from the SIMS analysis as shown in Figure 6d that Fe ions have been suppressed near the channel. We computed 2DEG sheet resistance (R_sh_) ~278 Ω/□ for C-doped buffer and ~269 Ω/□ for C/Fe-doped buffers. The slightly lower R_sh_ indicates no measurable channel compensation and supports our conclusion that Fe is effectively confined away from the channel. Consequently, the inferior DC/dynamic R_on_ of the C/Fe-doped buffers are not driven by the channel sheet conduction but are instead buffer-related (e.g., higher trap density), consistent stronger low-frequency noise, or of poor epitaxial quality. The R_on_ includes channel R_sh_, access/contact contributions, and a trap-induced dynamic term. Although R_sh_ is slightly lower, Fe-related deep traps and higher dislocation density in the composite buffer produce a virtual-gate effect and remote Coulomb scattering that locally deplete the 2DEG during biased operation, thereby lowering the effective mobility and increasing both drain lag and dynamic R_on_.

Higher C concentration (e.g., 1 × 10^20^ cm^−3^) can effectively suppress Fe diffusion [25]; however, the epitaxial growth of composite buffers remains challenging. In epitaxial quality analysis, (002) and (102) full width of half-maximum (FWHM) values were measured as 443/640 arcsec for the single C-doped buffer and 701/1140 arcsec for the C/Fe-doped buffer. It was observed that the FWHM values of the composite buffer were greater than those of the single C-doped buffer. This suggests that the ratio and concentration of C/Fe in the composite buffer still require further optimization. Trap-related dislocation densities were estimated based on the (002)/(102) FWHM values using the empirical formula [26,27]. The total dislocation of single C-doped buffer was 2.57 × 10^9^/cm^2^ and that of C/Fe-doped buffer was 7.89 × 10^9^/cm^2^. Although the composite buffers demonstrate promising control over Fe diffusion, the poor FWHM negatively impacts device characteristics. There must be a trade-off between Fe diffusion, trap concentration, and electrical performance. The increased defect density in the composite buffer is attributed to the incorporation of Fe, which introduces deep-level traps located at approximately 0.5–0.6 eV below the conduction band edge. These Fe-related traps help compensate intrinsic carriers and strengthen the semi-insulating effect, but also increase dislocation-related defects. Thus, the concentration and thickness of the Fe-doped buffer can be adjusted to improve the semi-insulating effect and further enhance breakdown voltage [28]. In contrast, the C-doped buffer contributes to acceptor states near 0.9 eV above the valence band with minimal disruption to crystal quality. Therefore, while the single C-doped buffer offers better epitaxial properties, the composite C/Fe-doped buffer achieves stronger carrier compensation, improved vertical breakdown voltage (Figure 6b) and semi-insulating properties, which is critical for high-power applications. It should be noted that the dislocation densities derived from the X-ray rocking curve FWHM represent an average defect density across the entire GaN buffer layer, rather than localized trap concentrations near the channel/C-doped GaN interface. To minimize defect-induced degradation, future optimization may involve reducing the Fe-doped buffer thickness or slightly lowering the Fe concentration. Since excessively increasing the C concentration could degrade epitaxial quality, alternative approaches such as periodically C-doped superlattices or double C-doped layer configurations may serve as effective strategies to compensate for lower Fe doping or limitations in increasing C concentration. These results highlight the essential trade-off between structural quality, trap concentration, and electrical isolation in GaN HEMT buffer engineering.

**Figure 7:**
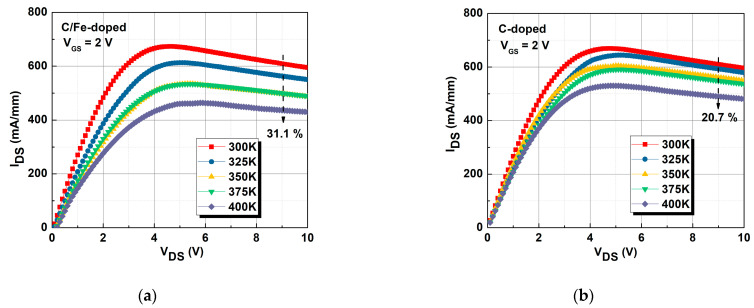
I_DS_-V_DS_ curves at different temperatures for the devices with (**a**) C/Fe-doped buffer and (**b**) single C-doped buffer.

Figure 7a,b show the curves of I_DS_ against V_DS_ for 300 to 400 K for the C/Fe-doped and single C-doped buffers. The maximum I_DS_ values of the device with the single C-doped buffer at 300 and 400 K were 595.72 and 480.8 mA/mm, respectively, with a decrease in current of 20.7%. The maximum I_DS_ values of the device with the C/Fe-doped buffer at 300 and 400 K were 594.66 and 429.94 mA/mm, respectively, with a decrease in current of 31.1%. The R_on_ values for the single C-doped buffer at 300 and 400 K were 4.21 and 5.39 Ω∙mm, respectively, an increase of 22%. The R_on_ values for the C/Fe-doped buffer at 300 and 400 K were 4.13 and 7.24 Ω∙mm, respectively, an increase of 42.9%. I_DS_ decreased with temperature due to decreased mobility in the channel, leading to increased resistance [29,30] for both devices. The higher thermal conductivity of the single C-doped buffer led to more effective heat dissipation and a smaller I_DS_ roll off than that exhibited by the C/Fe-doped buffer.

**Figure 8:**
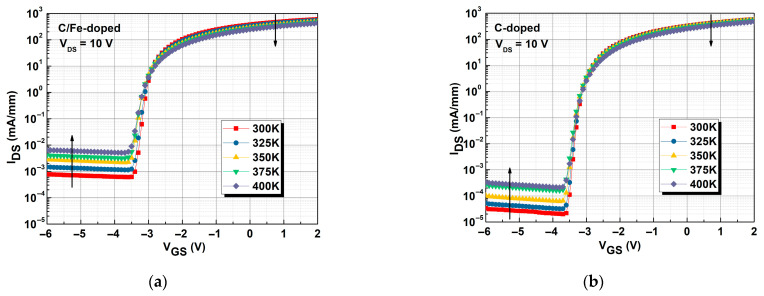
Subthreshold characteristics at different temperatures for the devices with (**a**) C/Fe-doped buffer and (**b**) single C-doped buffer.

Figure 8a,b show the subthreshold characteristics for 300 to 400 K for the C/Fe-doped and single C-doped buffers, respectively. On-state I_DS_ degradation was the result of lower 2DEG mobility, which in turn was caused by greater phonon scattering at higher temperatures, consistent with the results presented in Figure 7. The increase in off-state I_DS_ at higher temperatures was caused by electron leakage from the buffers [29]. The number of defects in the single C-doped buffer was lower than that in the C/Fe-doped buffer, as shown in Figure 4 and Figure 5. Thus, a lower number of electrons were released due to defects in the C-doped buffer compared with those in the C/Fe-doped buffer layer. Because the C-doped buffer had higher activation energy (and thus a higher energy barrier) [24], the electrons in the trap were not easily released at 300 K. However, the Fe-doped buffer had a lower energy barrier, resulting in greater electron release at 300 K. Therefore, the off-state I_DS_ was lower in the device with the C-doped buffer than that with the C/Fe-doped buffer.

Table 1 summarizes GaN HEMTs employing C-, Fe-, and composite C/Fe-doped buffers, together with parameters (substrate, passivation, and breakdown criteria) reported by other groups in recent years. Notably, our composite C/Fe buffer—implemented without a back barrier or passivation—still exhibits some potential, indicating meaningful headroom for device improvement. Thus, the composite architecture emerges as a tunable platform that broadens the GaN HEMT design space through mechanism-guided optimization of Fe dose and spatial placement, as well as the thickness/periodicity of C interlayers.

## 4. Conclusions

This study compared the effectiveness of a single C-doped buffer layer and a composite C/Fe-doped buffer layer in an AlGaN/GaN HEMT. The C-doped GaN buffer layer had fewer defects and better dynamic characteristics compared with the composite C/Fe-doped buffer layer, which was determined based on reduced leakage current characteristics, improved pulse measurement, and suppressed low-frequency noise, all of which are beneficial for applications in high-power devices. However, the composite C/Fe-doped buffer still shows significant potential for further development. Its dual-layer design provides a more tunable platform to balance semi-insulating behavior and dynamic performance, particularly in achieving lower channel R_sh_, enhanced vertical breakdown voltage, and stronger suppression of Fe-related diffusion effects. With proper optimization of doping concentrations and layer thicknesses, the composite buffer structure could offer a practical and scalable solution for next-generation GaN-based power devices.

## Figures and Tables

**Table 1:** Summary of current density of GaN HEMTs employing C-doped, Fe-doped, and C/Fe-doped buffers reported by other groups.

Buffer/Substrate	Maximum I_D_ (mA/mm)	Buffer Layer Thickness (µm)	Leakage Current Density (mA/mm)	Breakdown Voltage (V)	Reference
C-doped GaN/Si with passivation SiN	600	2	10^−6^ *	700 *, vertical	[31]
Fe-doped GaN/SiC with passivation SiO_2_	580	1.2	10^−7^ **	760 ** L_GD_ = 14 µm	[8]
UID/Fe/C-doped GaN/Si with back barrier and passivation Si_3_N_4_	1000	1.5	10^−7^ *	710 *, horizontal	[17]
Fe/C-doped GaN/SiC with back barrier and passivation SiN	990	1.2	10^−4^ **	>200 ** L_GD_ = 4 µm	[16]
C/Fe-doped GaN/Si without passivation	594	1.5	10^−4^ *, horizontal 10^−7^ *, vertical	690 *, horizontal 1055 *, vertical	This work
C-doped GaN/Si without passivation	583	1.5	10^−4^ *, horizontal 10^−6^ *, vertical	979 *, horizontal 1025 *, vertical	This work

The symbol * represents two-terminal breakdown voltage and leakage current. ** represents three-terminal breakdown voltage and leakage current.

## Data Availability

The original contributions presented in this study are included in the article. Further inquiries can be directed to the corresponding author.
